# Characterization of Taste Compounds and Sensory Evaluation of Soup Cooked with Sheep Tail Fat and Prickly Ash

**DOI:** 10.3390/foods11070896

**Published:** 2022-03-22

**Authors:** Yan Huang, Dandan Pu, Zhilin Hao, Li Liang, Jing Zhao, Yizhuang Tang, Yuyu Zhang

**Affiliations:** 1 Beijing Key Laboratory of Flavor Chemistry, Beijing Technology and Business University, Beijing 100048, China; huangyan_916@163.com (Y.H.); 18518351472@163.com (D.P.); hzl15716324037@163.com (Z.H.); gcfll@126.com (L.L.); yizhuang.tang@foxmail.com (Y.T.); 2 College of Food Science and Nutritional Engineering, China Agricultural University, Beijing 100083, China; zhaojing_cau@hotmail.com

**Keywords:** sheep tail fat, taste compound, spearman correlation analysis, additional experiment, sensory evaluation

## Abstract

Sheep tail fat and prickly ash play an important role in improving the umami taste of mutton soup. In this work, the effects of prickly ash on key taste compounds in stewed sheep tail fat soup were investigated. Results showed that the taste intensity of sheep tail fat soup cooked with 0.2% prickly ash increased significantly. The concentration of organic acids and free amino acids in sheep tail fat soup significantly increased with the addition of prickly ash. The concentration of succinic acid (2.637 to 4.580 mg/g) and Thr (2.558 to 12.466 mg/g) increased the most among organic acids and amino acids, respectively. Spearman’s correlation analysis elucidated that seven taste compounds were positively correlated (correlation coefficient > 0.7) with the overall taste intensity of the soup sample including Thr, Asp, oxalic acid, lactic acid, citric acid, succinic acid, and ascorbic acid. Additional experiments and quantitative descriptive analysis further confirmed that Asp, lactic acid and citric acid were the key taste compounds to improve saltiness and umami taste in sheep tail fat soup with prickly ash.

## 1. Introduction

Sunit sheep are farmed in the central part of the northwestern China area. The sheep tail fat accounts for about 10% of the body weight [1]. In addition, the fat content is more than 87% in sheep tail fat. High fat content promotes the characteristic flavor of mutton. However, there is less comprehensive utilization of sheep tail fat. Thus, it has brought a series of problems such as environmental pollution and resource waste.

Sheep tail fat is rich in flavor precursor substance, which is an important factor to generate the flavor. Studies have shown that the characteristic flavor of sheep meat is related to fat and fat oxidation [2]. Fat oxidation forms carbonyl compounds such as aldehydes, ketones and acids, which plays an important role in odor characteristics [3]. In the thermal processing, the fat-soluble flavor substances contained in fat cells are released, which contribute to the enhancement of meat flavor [4]. In addition, the Maillard reaction is also crucial for the formation of meat flavor. The addition of glycine (Gly) was conducive to the formation of meat flavor in the Maillard reaction model [5]. Moreover, the addition of fat changes the type and quantity of Maillard reaction products, and the synergistic effect of lipid oxidation and Maillard reaction also produce some new reaction products [6].

In northwest China, sheep tail fat functions as a food condiment when cooking meals. A previous study reported that water-soluble compounds have a significant contribution to taste [7]. Stewing may help the release of these taste compounds to their maximum extent [8]. Most researchers focus on umami taste in the research of meat products, free amino acids and nucleotides are important taste related compounds in meat products [9]. Due to the different structure of amino acids, they exhibit different taste characteristics, which are mainly umami, sweetness and bitterness. Aspartic acid (Asp) and glutamic acid (Glu) are the typical representatives of umami taste [10]. In addition, with the participation of inosine 5′-monophosphate (5′-IMP) and guanosine 5′-monophosphate (5′-GMP), the umami taste is significantly enhanced. In different food systems, the addition of organic acids will bring different flavor changes, which relate to the content and types of organic acid addition. Kang et al. found that adding lactic acid to beef soup could enhance the sourness, saltiness and beefy taste. Taste compounds added to different food systems also present different taste characteristics due to the complex interactions of different components [11]. Hence, the key taste compounds need to be characterized in different systems [12].

The addition of spices represents an effective way to enhance the overall taste, as widely used in meat processing industry [13]. Moreover, spices have the function of bacteriostasis and antisepsis [14]. Spices generally bear their own unique flavor compounds [15,16], and when added to meat products, they improve the overall taste. On the production of sheep meat sausages, chili powder was added to inhibit or cover the unpleasant flavor of sheep meat, so as to improve the overall taste [17]. Wang et al. also reported that mixed spices could promote the release of free amino acids in stewed beef soup, and therefore, significantly increase the equivalent umami concentration (EUC) of stewed beef soup [18]. Moreover, prickly ash (*Zanthoxylum bungeanum* Maxim) is popular and widely used in the meat processing. Xi et al. found that the addition of spices can modify flavor and partially neutralize unpleasant odors present in roast sheep meat, and prickly ash improved the content of terpenes greatest [19]. The prickly ash is rich in flavonoids and amides, which has been reported with bitter taste [20] and pungent sensation [21], respectively. This may be related to the fact that prickly ash improves the overall taste of meat products.

The aims of this work were to investigate the prickly ash and its taste contribution of stewed sheep tail fat: (1) Determination of free amino acids, 5′-nucleotides, and organic acids in stewed sheep tail fat (soup, fat block and upper oil) samples by high-performance liquid chromatography (HPLC); (2) Prediction of the correlated taste compounds to sensory properties by Spearman’s correlation analysis; (3) Confirmation of the contribution of the predicted taste compounds to taste perception by additional experiments and quantitative description analysis (QDA).

## 2. Materials and Methods

### 2.1. Materials and Chemicals

Sheep tail fat (Sunit sheep) was purchased from Xilingol league, Inner Mongolia. Dried ginger (Shandong Province), cassia (Guangxi Province), nutmeg (Guangdong Province), white pepper (Guangxi Province) and prickly ash (Guangxi Province) were purchased from Yonghui supermarket, Beijing. Oxalic acid, ascorbic acid, citric acid, succinic acid, lactic acid, hydrochloric acid (HCl) and phosphoric acid (H_3_PO_4_) were purchased from Sinopharm Chemical Reagent Co. (Shanghai, China). Cytidine 5′-monophosphate (5′-CMP), 5′-GMP, 5′-AMP and 5′-IMP were purchased from Sigma-Aldrich (St. Louis, MO, USA). Monopotassium phosphate (KH_2_PO_4_), sodium dihydrogen phosphate dodecahydrate (Na_2_HPO_4_·12H_2_O) and sodium borate decahydrate (Na_2_B_4_O_7_·10H_2_O) were purchased from Alfa Aesar (Ward Hill, MA, USA). All these chemicals are analytical grade with purity ≥ 98%. HPLC grade methanol, acetonitrile and isopropanol were purchased from Fisher Scientific (Shanghai, China). Ultra-pure water was supplied by Hangzhou Wahaha Group Co., Ltd. (Hangzhou, China).

### 2.2. Sample Preparation

Based on a preliminary test [18], four factors were selected including solid–liquid ratio (1:1, 1:2, 1:3, 1:4 and 1:5), stew time (1.0, 1.5, 2.0, 2.5 and 3.0 h), spice selection (ginger, cassia, nutmeg, white pepper and prickly ash), and the content of optimized spice addition (0.1%, 0.2%, 0.3%, 0.4% and 0.5%). Each optimal factor was determined by the results of sensory evaluation, and more information about four factors is listed in Table S1.

Preparation of stewed sheep tail fat with prickly ash (SSP): sheep tail fat (100 g), water (200 g) and optimized spice powder (0.60 g) were mixed and stewed for 3 h in a stew pan. After stewing, fat block and the liquid were separated, the liquid was further filtered to remove impurities. After standing for about 20 min at 4 ℃, the water phase (soup) and the oil phase (upper oil) of the filtered liquid were separated. The soup sample was centrifugated (10,000 rpm) for 10 min at 4 ℃ for further analysis and labeled as SSP-S. The upper oil was mixed with 50% edible ethanol at a ratio of 1:1 (*w*/*w*) and treated with ultrasound at 400 W for 15 min (Kunshan Ultrasonic Instrument Co., Ltd., Kunshan, China). After ultrasonic treatment, the sample was filtered and the filtrates were transferred to a separating funnel. When the aqueous phase and oil phase were separated, the aqueous phase sample was collected and labeled as SSP-O. Similar as described above, stewed fat block was placed in a beaker and mixed with 50% edible alcohol at a ratio of 1:1 (*w*/*w*) before ultrasonicating for 30 min at 400 W. After ultrasonic treatment, the sample was filtered with a gauze and the filtrates were transferred to a separating funnel. When the aqueous phase and oil phase were separated, the aqueous phase sample was collected and labeled as SSP-B.

Preparation of stewed sheep tail fat (SS): sheep tail fat (100 g) and water (200 g) were mixed and stewed for 3 h in a stew pan. After stewing, the same procedure as described above was used to obtain the soup sample (SS-S), fat block sample (SS-B) and upper oil sample (SS-O).

Preparation of stewed optimized spice (SP): the selected spice powder (0.60 g) and water (200 g) were mixed and stewed for 3 h in a stew pan. After stewing, the solids were removed by centrifugation to obtain the sample.

### 2.3. Sensory Analysis

Sensory evaluation: Before taste evaluation, all panelists knew sample information, and taste evaluation tests were conducted according to the Helsinki Declaration [22,23] Moreover, sensory evaluation was approved of the Ethics Committee of China Agricultural University (CAUHR2021022, Beijing, China) [24]. All panelists have received basic taste training of sourness, sweetness, bitterness, saltiness and umami, and have a certain understanding of the taste information about sheep tail fat. A total of 12 panelists (5 males and 7 females, aged 20–30) were selected for the taste evaluation.

The taste evaluation (ranking method) of stewed sheep tail fat (four factors) was carried out by GB/T 12315-2008, China Standard Press. The rank sum of each sample was obtained by adding the overall taste ranks of panelists (5 represents the best overall acceptance; 1 represents the lowest overall acceptance). The differences between samples were distinguished according to the Friedman test (*F*_test_) and least significant difference (LSD) value.

QDA was used to verify the saltiness and umami enhancement effect of the compounds. Firstly, the taste thresholds of sodium chloride (saltiness) and monosodium glutamate (umami) solutions were evaluated. The panelists evaluated a series of concentration gradients of sodium chloride and monosodium glutamate solutions (10.00, 5.00, 2.00, 1.00, 0.50, 0.10, 0.05, 0.01 g/L). In addition, the threshold concentration was obtained according to whether 50% of the panelists could perceive the corresponding taste. Different concentrations of compounds were added to the threshold concentration of sodium chloride and monosodium glutamate solution. Saltiness and umami enhancement effects were evaluated by sensory score (1–9, 1 is low and 9 is high). The addition concentration of compounds were listed in Table S2.

### 2.4. Electronic Tongue

Soup samples including SS-S and SSP-S were analyzed by SA402B (Insent, Japan) electronic tongue system. Before detection, five sensors including CA0 (sourness), AAE (umami), C00 (bitterness), CT0 (saltiness), and GL1 (sweetness) were pretreated in a reference solution (30 mM KCl solution containing 0.3 mM tartaric acid) for 24 h before analysis. Instrument needs to process auto-checked procedure to ensure the stability and reliability of the instrument. The detection procedure was set as follows: sample taste collection time (30 s), aftertaste collection time (30 s) and cleaning time (300 s). In addition, the test was conducted at room temperature of 25 °C. After detection, potential information was converted into taste information for further analysis.

### 2.5. HPLC Analysis

Determination of organic acids: organic acids were determined by following a reported method [15,16]. HPLC detection conditions are as follows: instrument, Thermo U3000 (Thermo Scientific, Waltham, MA, USA); chromatographic column, Venusil MP C18 (4.6 mm × 250 mm, 5 μm); mobile phase A, 0.01 mol/L KH_2_PO_4_ (pH 2.8 with H_3_PO_4_); mobile phase B, methanol; equivalent elution with 95% mobile phase A and 5% mobile phase B; flow rate, 1.0 mL/min; injection volume, 20 μL; column temperature, 25 °C; detection time, 20 min; diode array detector (DAD) with the detection wavelength of 205 nm.

Determination of 5′-nucleotides [15,16]: The mobile phase A, mobile phase B, detection time and detection wavelength were 0.05 mol/L KH_2_PO_4_, methanol, 15 min, and 254 nm, respectively. Other detection conditions of HPLC for 5′-nucleotides were the same as the organic acid.

Determination of free amino acids: The determination of free amino acids was based on the method of [15,16] with minor optimization. The sample (2 mL) was diluted to 5 mL using 0.1 mol/L hydrochloric acid. The diluted sample was placed in the dark for 1 h before centrifuging at 4 °C for 10 min. After centrifugation, the supernatant was taken to obtain the analyzed sample. Durashell AA reagent analysis kits (Tianjin Bona Agel Technology Co., Ltd., Tianjin, China) were used for the analysis of free amino acids. A 500 μL sample and 50 μL amino acid internal standard solution was mixed for injection analysis. Agilent 1260 (Agilent Corp., Karlsruhe, Germany) was used to detect free amino acids. The detection conditions were as follows: Mobile phase A, 9.5 g Na_2_B_4_O_7_·10H_2_O and 9 g Na_2_HPO_4_·12H_2_O dissolved in 2 L ultrapure water, and pH was adjusted to 8.2 by HCl before filtration; Mobile phase B, 45% (*V*/*V*) acetonitrile, 45% (*V*/*V*) methanol and 10% (*V*/*V*) ultrapure water mixed; chromatographic column, Venusil Durashell AA (4.6 mm × 150 mm, 3 μm); column temperature, 45 °C; DAD detection wavelengths, 338 and 262 nm; the elution gradient was as follows: 0–6 min, 6–10% B; 6–8 min, 10% B; 8–10 min, 10–16% B; 10–23 min, 16–40% B; 23–30 min, 40–50% B; 30–31 min, 50–100% B; 31–34 min, 100% B; 35–38 min, 6% B; the flow rate, injection volume and detection time were 1.6 mL/min, 20 μL and 38 min, respectively.

### 2.6. Calculation of EUC

EUC (g MSG/100 g) refers to 100 g sample that can be equal to the mass of sodium glutamate, which relates to umami amino acids (Asp and Glu) and 5′-nucleotides (5′-IMP, 5′-GMP and 5′-AMP). It can be calculated by the following equation [18]:

EUC (g/l00 g) = Σa_*i*_ × b_*i*_ + 1218 (Σa_*i*_ × b_*i*_) (Σa_*j*_ × b_*j*_) (1)

where, a*_i_* is the concentration (g/100 g) of each umami amino acid (e.g., Asp or Glu); b*_i_* is the relative umami concentration for each umami amino acid (Glu, 1 and Asp, 0.077); a*_j_* is the concentration (g/100 g) of umami 5′-nucleotide (5′-IMP, 5′-GMP or 5′-AMP); b*_j_* is the concentration for each umami 5′-nucleotide (5′-IMP, 1; 5′-GMP, 2.3 and 5′-AMP, 0.18), and 1218 is a synergistic constant based on the concentration of g/100 g.

### 2.7. Additional Experiments

In order to investigate the taste enhancing effect of compounds added to the sample of SS-S, additional experiments were conducted for analysis, and electronic tongue was used for taste verification [25,26]. The concentration of the added compounds was determined by SSP-S sample minus the content in SS-S sample. According to previous work, a total of seven compounds were obtained including AD1: Thr, 153.61 mg/L; AD2: Asp, 3.99 mg/L; AD3: oxalic acid, 2.73 mg/L; AD4: lactic acid, 3.29 mg/L; AD5: citric acid, 0.93 mg/L; AD6: succinic acid, 31.10 mg/L; AD7: ascorbic acid, 0.25 mg/L. Moreover, the seven compounds were analyzed by electronic tongue with their corresponding concentrations (SC1-7).

### 2.8. Statistical Analysis

The value of *F*_test_ was calculated based on Equation (2) and compared with the F critical value (a value determined by the number of samples and evaluators). Based on *F*_test_ > F, LSD was calculated according to the Equation (3) to determine which samples were significantly different from other samples in groups.

(2) $$F_{\mathrm{test}}=\frac{12}{\mathrm{jp}(p+1)}\left(R_{1}^{2}+\ldots +R_{P}^{2}\right)-3j\left(p+1\right)$$

(3) $$\mathrm{LSD}=z\sqrt{\frac{\mathrm{jp}(p+1)}{6}}$$

Note: *j*, number of sensory panelists; *p*, number of samples; *Rp*, rank sum of *p* samples.

Spearman’s correlation coefficient analysis (*R* package) was considered significant at *p* < 0.05. Human sensory evaluation, EUC and taste compounds were used for correlation analysis (only in SS-S and SSP-S samples). Positive correlation (correlation coefficient > 0.7) between sensory evaluation and taste compounds was found, and additional experiments were used to verify the taste characteristics of the compounds.

The electronic tongue signal was collected through Taste sensing system application (Insent, Japan). In addition, the potential value was converted into taste value for further analysis through Taste analysis system application (Insent, Japan). Taste compounds were performed in triplicate and the results were expressed as the mean ± standard deviation (SD). The data were analyzed by a one-way analysis of variance and Duncan’s multilevel tests (*p* < 0.05) by SPSS software (version 25.0, IBM, New York, NY, USA). The linear relationship between compound concentration and saltiness/umami intensity was analyzed by Excel (Microsoft Co., Ltd., Washington, DC, USA).

## 3. Results and Discussion

### 3.1. Spices Addition Results

**Figure 1:**
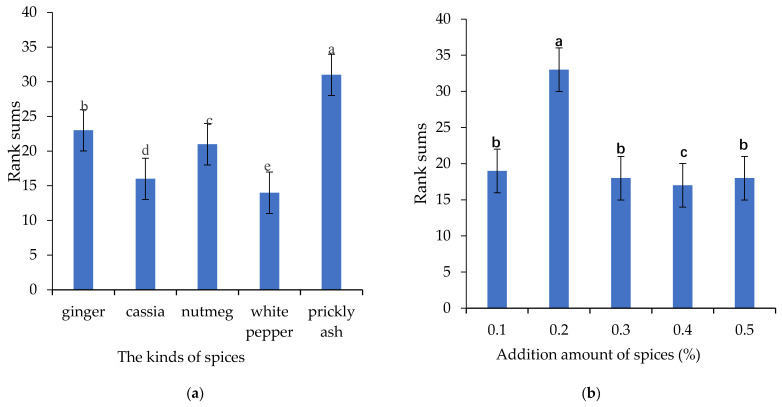
Results of spices selection and addition ((**a**), rank sums of five spice varieties; (**b**), rank sums of addition ratio from 0.1 to 0.5%). Letters (a, b, c) indicate significantly difference at *p* < 0.05 (Duncan test).

The results of solid–liquid ratio and stewing time of stewed sheep tail fat are shown in Figure S1. According to the results of sensory evaluation, the solid–liquid ratio and stewing time were optimized to be 1:2 and 3 h, respectively. Furthermore, the selection and addition of spices were optimized (Figure 1). This experiment assessed 5 spices (e.g., ginger, cassia, nutmeg, white pepper and prickly ash) that are commonly used in daily household with stewing meat products. Prickly ash shows the best overall taste intensity with single factor optimization experiment (Figure 1a). Spices have unique flavor characteristics, which can cover up the off-flavor (e.g., blood smell and stinky) of meat products, and cooperate with some compounds in meat to improve the overall taste of meat products. Spices are rich in flavonoids, and can exhibit antioxidant, antibacterial and antiseptic properties, when processed together with meat products [27]. Yu et al. evaluated the effects of spices on flavor compounds in the stewing process of Wuding chicken. The results showed that the addition of spices increased the water-soluble low molecular compounds, and exhibited a significant effect on the release of volatile compounds [28].

The optimization results of adding amount were shown in Figure 1b. Different amounts (0.1%, 0.2%, 0.3%, 0.4%, 0.5%, percentage of the total weight of sheep tail fat and water) of prickly ash were added with stewing sheep tail fat. Previous study reported that prickly ash could improve the flavor of meat products [29]. Lower amount of prickly ash addition could not improve the flavor of meat products; and the taste of prickly ash would cover up the taste of meat products with higher addition. 0.2% prickly ash addition was potimized with the best overall taste intensity.

### 3.2. Quantification of Taste Compounds

Free amino acids can be devided into four categories according to their taste characteristics [30,31]: umami amino acids (Asp and Glu), sweetness amino acids(serine (Ser)), alanine (Ala), Gly, threonine (Thr)), bitterness amino acids (arginine (Arg), histidine (His), tyrosine (Tyr), leucine (Leu), valine (Val), methionine (Met), isoleucine (Ile), phenylalanine (Phe), lysine (Lys), proline (Pro), and tasteless amino acid (cysteine (Cys)). Free amino acids in the samples were quantified by pre-column derivatization liquid chromatography method. The final quantitative results are shown in Table 1. The proportion of three types of free amino acids (umami, sweetness, bitterness) are shown in Figure S2. After adding prickly ash, the total sweet amino acids in soups increased from 50% (SS-S) to 56% (SSP-S). The addition of prickly ash could significantly promote the release of Thr in the soup samples, from 2.558 mg/g (SS-S) to 12.466 mg/g (SSP-S). However, the total sweet amino acid content in fat block samples reduced from 79% (SS-B) to 20% (SSP-B) with the addition of prickly ash. The content of Thr was 4.161 mg/g in SS-B sample and 1.019 mg/g in SSP-B sample. Combined with soup samples, it was speculated that the content of Thr in stewed sheep tail fat without the addition of prickly ash was not released completely with higher content in stewed fat block. Prickly ash could promote the release of Thr in soup samples. This result is similar with the result of Wang et al. [32]. The total bitter amino acids in the soup decreased from 46% (SS-S) to 41% (SSP-S) after adding prickly ash. However, the content of total sweet amino acids has no significant change, 30.359 mg/g in SS-S sample and 30.698 mg/g in SSP-S sample. Phe and Ile are the most bitter amino acids in soup samples, ranging from 9.144 to 10.702 mg/g, which played an important role in the taste of soup samples. Umami amino acids are the most important in meat products. With adding prickly ash, Asp increased from 0.511 mg/g to 0.755 mg/g, and Glu decreased from 1.953 mg/g to 1.765 mg/g. Although the Glu content decreased after adding prickly ash, it promoted the release of Asp in fat block samples (1.011 mg/g) and upper oil samples (0.500 mg/g).

The content of 5′-nucleotides in soup samples, fat blocks samples and upper oil samples was shown in Table 1. The 5′-nucleotides represent a kind of important taste compounds. Three 5′-nucleotides, including 5′-CMP, 5′-GMP and 5′-IMP, were detected in samples. Only 5′-CMP was detected in SP sample, with a content of 0.010 mg/g, which related to the low concentration of stewed prickly ash. The nucleotides 5′-CMP and 5′-IMP were only detected in soup samples (SSP-S and SS-S). Meanwhile, 5′-GMP was detected in all six samples. 5′-GMP exists in the organic phase of water-in-fat emulsions [33]. It is important to note that processing technology could not completely release the taste compounds, and some compounds still have a certain content in the processed meat products. The content of 5′-GMP decreased from 0.074 mg/g to 0.044 mg/g, and 5′-IMP increased from 0.090 mg/g to 0.142 mg/g, after adding prickly ash.

The main taste characteristics of organic acids are sournes. Other taste characteristics, such as sodium succinate, has umami or umami enhancing effect [22,34]. In Table 1, a total of five organic acids (oxalic acid, ascorbic acid, lactic acid, citric acid and succinic acid) were detected in soups, fat blocks and upper oils samples. The addition of prickly ash promoted the release of organic acids in soup samples, and the contents of five kinds of organic acids in the stewed fat block and upper oil decreased (except for succinic acid in the stewed fat block samples). The addition of prickly ash promoted the release of organic acids in the soup samples. Moreover, organic acids also existed in prickly ash, the content of organic acids in SSP-S sample increased with an additive effect. Oxalic acid was detected in all samples, and the content of oxalic acid in soup samples was higher, measuring 0.727 mg/g in SS-S sample and 0.883 mg/g in SSP-S sample. Oxalic acid was present in stewed sheep tail fat, which may due to the fact that oxalic acid is not metabolized in the body after sheep eat an abundance of plant-based food [35]. The content of succinic acid was the highest in soup samples, 2.637 mg/g in SS-S sample and 4.580 mg/g in SSP-S sample.

### 3.3. Analysis of EUC

The result of EUC was shown in Figure S3. The EUC values in soup samples were higher, SS-S was 7.748 g MSG/100 g, SSP-S was 4.474 g MSG/100 g. The EUC values of stewed fat block and upper oil ranged from 0 to 0.386 g MSG/100 g, which was significantly lower than soup samples. This may be because taste compounds were mainly water soluble compounds [36], thus they were released in soup samples. The addition of prickly ash reduced the EUC value in soup samples, as the content of 5′-nucleotides and Glu in the soup samples decreased after the addition of prickly ash.

### 3.4. Correlation Analysis

**Figure 2:**
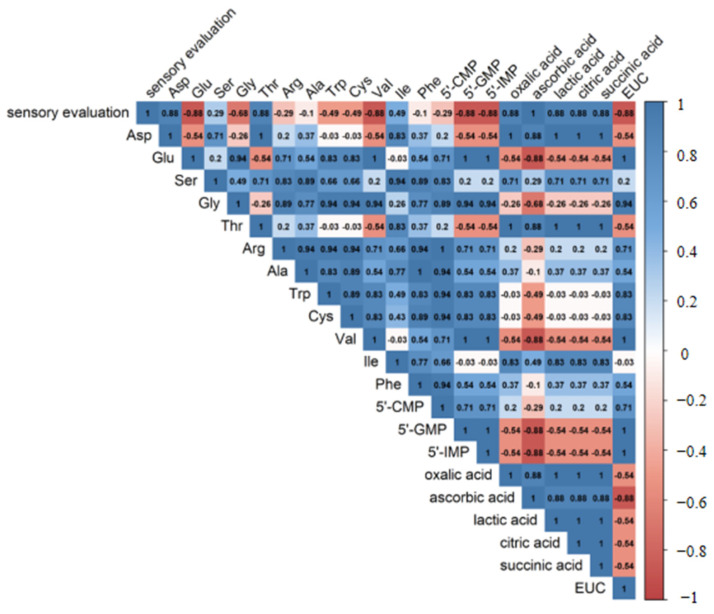
The results of Spearman correlation analysis.

Spearman’s correlation analysis is shown in Figure 2. The correlation relationship was considered significant when *p* < 0.05 and correlation coefficient > 0.7 [37]. The results showed that seven compounds including Thr, Asp, oxalic acid, lactic acid, citric acid, succinic acid and ascorbic acid had a significant positive correlation with sensory evaluation results. Therefore, these compounds were predicted as the potential taste-active compounds contributing to the taste perception of stewed sheep tail fat.

### 3.5. Electronic Tongue Analysis

**Figure 3:**
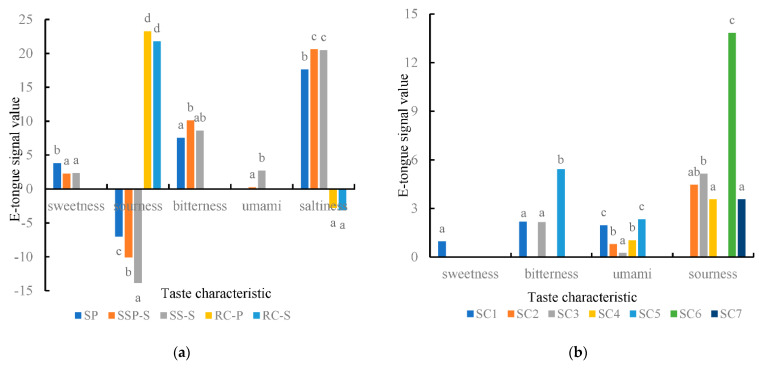

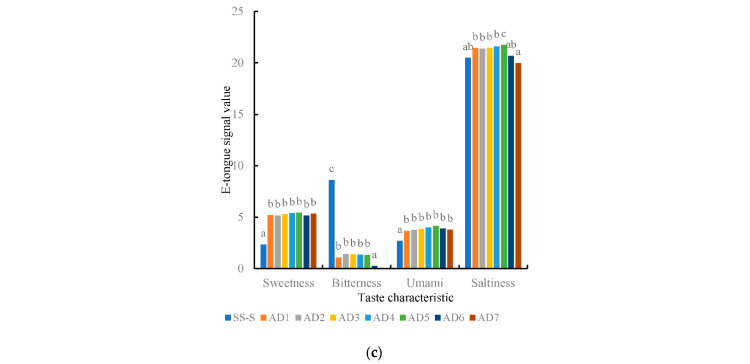
Electronic tongue analysis (**a**) analysis of different stewed samples; (**b**) analysis of standard compounds; (**c**) analysis of additional experiment). Letters (a, b, c) indicate significantly difference at *p* < 0.05 (Duncan test).

The electronic tongue analysis results (Figure 3a) showed that the bitterness of stewed sheep tail fat increased and umami taste decreased after adding prickly ash. SP sample was rich in flavonoids, and multivariate data analysis showed that they contributed to the bitter taste [20]. These flavonoids might be the reason for the enhancement of the bitter taste in SSP-S samples. Moreover, the addition of prickly ash reduced the umami taste in SSP-S samples, which might relate to the taste interaction. Bitterness taste played an important role in flavor contribution by regulating the taste of different foods. This result is similar to research conducted in cooked meat products by electronic tongue [38].

Seven correlated compounds were recombined according to their contents in samples SS-S and SSP-S. The recombinant samples obtained were labeled as RC-S and RC-P, respectively. These samples were analyzed by electronic tongue (Figure 3a). The main taste of the recombinant samples was sourness, which could ascribe to the properties of the compounds (five organic acids, one acidic amino acid and one neutral amino acid). Electronic tongue analysis of standard compounds were mainly sourness, bitterness and umami (Figure 3b). SC2, SC3, SC4, SC6 and SC7 had sourness taste characteristics, and SC6 was sourer, which could be due to the addition content of SC6 (31.10 mg/L). SC1, SC3 and SC5 had bitterness taste characteristics and SC1, SC2, SC4 and SC5 had umami taste characteristics. These compounds had rich taste characteristics, and could improve the overall taste characteristics when added to stewed sheep tail fat.

### 3.6. Additional Experiments

In order to verify the contribution of compounds to the taste of stewed sheep tail fat, samples AD1-7 were obtained by additional experiments, and electronic tongue results were shown in Figure 3c. Some compounds such as SC2, SC3, SC4 and SC6 had a certain sourness taste, and they did not increase the sourness value when they added to stewed sheep tail fat, but improved the umami and saltiness value. This may be because some sourness compounds could change the umami taste of samples. Ma et al. established fitting curves of relative umami intensity (sodium succinate) under different pH conditions. They found that when the pH of the sodium succinate solution was at 6.0, 6.5, and 7.0, the equivalent MSG concentration did not show much change; however, when pH increased from 7.5 to 8.0, the EUC decreased from 0.27 to 0.24 g/100 mL [39]. The taste interaction between sourness and umami may promote the umami taste. While, the effect of sourness compounds on the promotion of umami taste was only in a specific concentration range. When the concentration was low, there may be no promotion effect, and when the concentration was high, the existence of sourness taste may mask the umami taste. Miranda et al. evaluated the taste promotion effect of different concentrations of citric acid on constant concentrations of sodium glutamate. Participants were genotyped for the single nucleotide polymorphism rs34160967 in the TAS1R1 gene. The results showed that the perception of the intensity of umami taste decreased significantly as the concentration of sourness taste increased [40]. Some compounds have umami taste characteristics, such as Thr, Asp, lactic acid and citric acid, which also enhance the umami taste in SS-S samples through a synergistic effect.

### 3.7. Taste Evaluation of Compounds

The taste thresholds of sodium chloride and monosodium glutamate were obtained from panelists in laboratory with 0.71 and 0.22 g/L, respectively. Different concentrations of compounds (1, 2, 5, 7, and 10 times) added to solution of threshold concentration are listed in Table S2.

**Figure 4:**
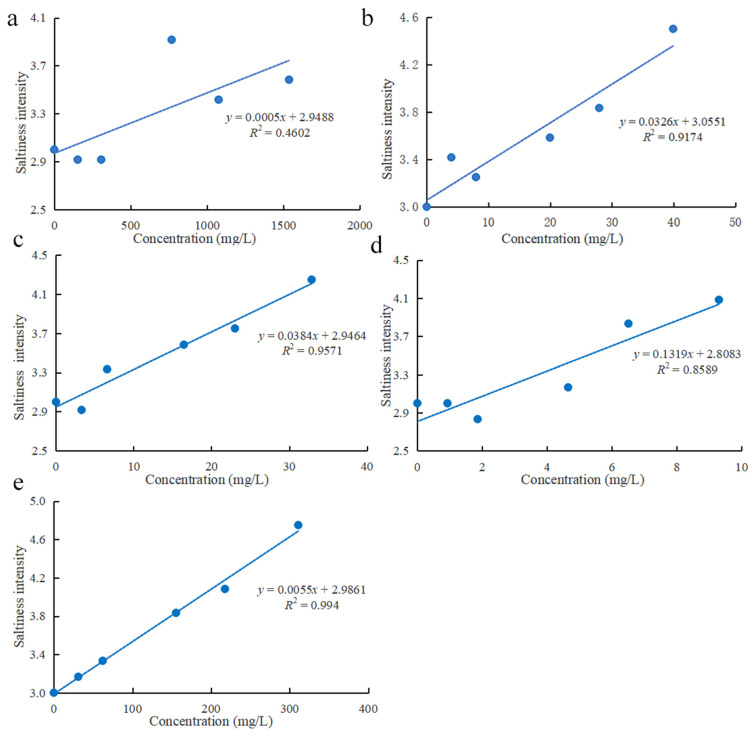
Sensory results of compounds added to sodium chloride solution (**a**) Thr; (**b**) Asp; (**c**) lactic acid; (**d**) citric acid; (**e**) succinic acid.

The analysis results of electronic tongue showed that these compounds had effects on umami taste and saltiness enhancement. To verify the accuracy of electronic tongue analysis results, the relationship between compounds concentration and saltiness/umami intensity was analyzed. The results of saltiness enhancing effect are shown in Figure 4. Compounds with *R*^2^ > 0.8 showed good linear correlation with taste evaluation, except for Thr with *R*^2^ = 0.4602. This might be because the taste intensity increased first and then decreased due to the concentration increase of Thr. Besides, the concentration of Thr (768.05 mg/L) showed the highest intensity (Figure 4a). Thr is a sweetness amino acid, and can enhance the perception of saltiness at low concentrations [40]. With its concentration increased, the perception of saltiness was inhibited. Asp (Figure 4b) and succinic acid (Figure 4e) exhibited better saltiness promotion performance with highest taste evaluation scores of 4.50 and 4.70, respectively. This may be due to the fact they are umami enhancers. To a certain extent, saltiness and umami taste share a great similarity. Researchers usually use umami compounds or umami enhancing compounds to replace salt to achieve the effect of salt reduction. Hayabuchi et al. elucidated that umami perception can compensate for the loss of palatability caused by salt reduction. Moreover, the addition of an appropriate amount of an umami substance can facilitate salt reduction from 0.9 to 0.3% without sacrificing palatability [41]. Although citric acid (Figure 4d) and lactic acid (Figure 4c) did not have a higher taste perception, yet the slope value with 0.1319 and 0.0384 indicated that they have great potential for improving the saltiness taste.

**Figure 5:**
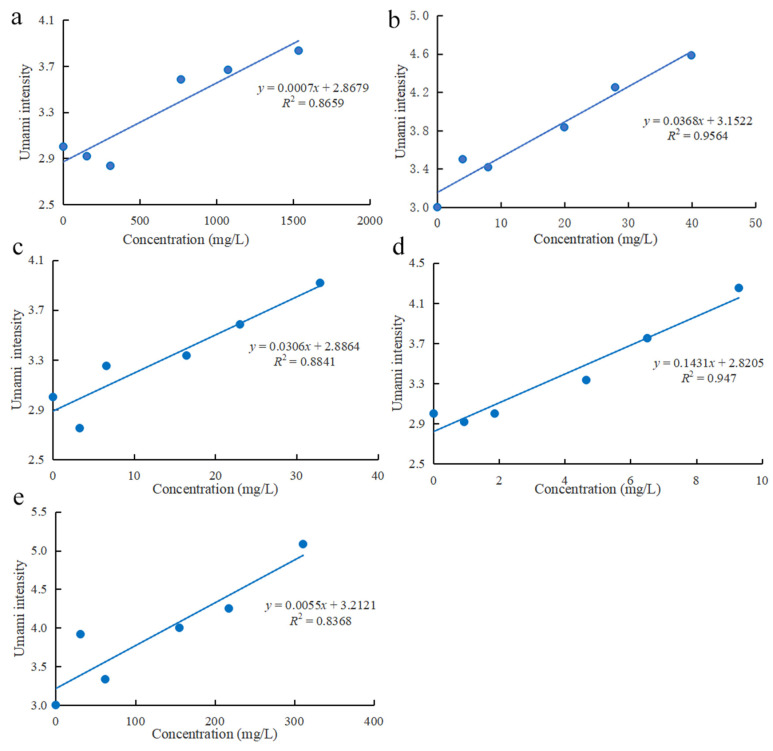
Sensory results of compounds added to monosodium glutamate solution (**a**) Thr; (**b**) Asp; (**c**) lactic acid; (**d**) citric acid; (**e**) succinic acid.

The umami enhancing effect results (Figure 5) showed that compounds with *R*^2^ > 0.8 showed good liner correlation with taste evaluation, which is similar to the results [32]. Asp (Figure 5b) and succinic acid (Figure 5e) were common umami enhancers, which are widely used in the food industry. They both showed a better effect on umami enhancement with the taste evaluation scores of 4.58 and 5.08, respectively. Lactic acid (Figure 5c) and citric acid (Figure 5d) also have an umami enhancing effect, which may be due to changes in pH [34] or taste interactions [42]. Moreover, citric acid showed the highest intercept value with 0.1431, which is the same as the saltiness enhancing effect.

In general, there was a good linear correlation between the concentration of five compounds and the sensory scores, which further demonstrated the accuracy and reliability of the electronic tongue results. Moreover, Asp and succinic acid showed a strong effect on the enhancement of umami and saltiness taste, which may be related to higher addition concentration and umami enhancer.

## 4. Conclusions

The optimization results of stewed sheep tail fat was 0.2% prickly ash addition, solid–liquid ratio 1:2 and stewing time 3 h. In addition, a total of twelve free amino acids, three 5′-nucleotides, and five organic acids were detected in stewed samples (soups, fat blocks and upper oils). Quantitative results showed that taste compounds were mainly released in soup samples, and the addition of prickly ash contributed to the release of taste compounds. Spearman analysis revealed that two free amino acids (Thr and Asp) and five organic acids (oxalic acid, lactic acid, citric acid, succinic acid and ascorbic acid) were positively correlated (with correlation coefficient > 0.7) with overall taste. Asp, lactic acid, and citric acid were the key taste compounds according to the results of additional experiments and QDA. The saltiness and umami taste of stewed sheep tail fat soup were enhanced with the addition of prickly ash. This work showed the contribution of prickly ash to the taste of stewed sheep tail fat soup, and provided theoretical support for the processing of sheep tail fat.

## Figures and Tables

**Table 1:** The content of free amino acids, 5′-nucleotides and organic acids in stewed sheep tail fat samples.

Compounds		Content (mg/g)						
		SP	SS-S	SSP-S	SS-B	SSP-B	SS-O	SSP-O
Umami	Asp	0.181 ± 0.002 ^a^	0.511 ± 0.013 ^b^	0.755 ± 0.045 ^c^	nd	1.011 ± 0.038 ^c^	nd	0.500 ± 0.019 ^b^
	Glu	0.061 ± 0.001 ^a^	1.953 ± 0.029 ^d^	1.765 ± 0.075 ^c^	0.262 ± 0.001 ^b^	0.301 ± 0.025 ^b^	nd	nd
Total umami amino acids		0.242 ± 0.003	2.464 ± 0.042	2.520 ± 0.120	0.262 ± 0.001 ^b^	1.312 ± 0.063	nd	0.500 ± 0.019
Sweetness	Ser	0.153 ± 0.002 ^a^	7.236 ± 0.381 ^c^	7.494 ± 0.290 ^c^	nd	nd	0.891 ± 0.019 ^b^	nd
	Gly	nd	8.606 ± 0.498 ^b^	7.771 ± 0.502 ^a^	nd	nd	nd	nd
	Ala	nd	14.887 ± 0.465 ^a^	14.828 ± 1.667 ^a^	nd	nd	nd	nd
	Thr	nd	2.558 ± 0.309 ^b^	12.466 ± 0.488 ^d^	4.161 ± 0.310 ^c^	1.019 ± 0.018 ^a^	nd	nd
Total sweetness amino acids		0.153 ± 0.002	33.287 ± 1.653	42.559 ± 2.947	4.161 ± 0.310	1.019 ± 0.018	0.891 ± 0.019	nd
Bitterness	Phe	nd	9.162 ± 0.180 ^b^	9.144 ± 0.214 ^b^	nd	1.984 ± 0.057 ^a^	nd	nd
	Arg	0.392 ± 0.002 ^a^	2.5480 ± 0.204 ^c^	2.464 ± 0.193 ^c^	0.822 ± 0.011 ^b^	0.823 ± 0.017 ^b^	nd	nd
	Val	0.064 ± 0.001 ^a^	5.841 ± 0.199 ^c^	5.152 ± 0.230 ^b^	nd	nd	nd	nd
	Tyr	nd	3.414 ± 0.205 ^b^	3.236 ± 0.111 ^a^	nd	nd	nd	nd
	Ile	nd	9.394 ± 0.496 ^a^	10.702 ± 1.710 ^b^	nd	nd	nd	nd
Total bitterness amino acids		0.456 ± 0.003	30.359 ± 1.284	30.698 ± 2.458	0.822 ± 0.011	2.807 ± 0.074	nd	nd
Tasteless	Cys-Cys	nd	5.651 ± 0.127 ^e^	5.452 ± 0.222 ^d,e^	4.884 ± 0.188 ^c^	5.31 ± 0.177 ^d^	4.396 ± 0.152 ^b^	4.054 ± 0.129 ^a^
Total amino acids		0.851 ± 0.008	71.761 ± 3.106	81.229 ± 5.747	10.129 ± 0.510	10.448 ± 0.332	5.287 ± 0.171	4.554 ± 0.148
	5′-CMP	0.010 ± 0.000 ^a^	0.188 ± 0.020 ^b^	0.177 ± 0.030 ^b^	nd	nd	nd	nd
	5′-GMP	nd	0.074 ± 0.018 ^b^	0.044 ± 0.002 ^a^	0.031 ± 0.002 ^a^	0.033 ± 0.005 ^a^	0.025 ± 0.016 ^a^	0.036 ± 0.018 ^a^
	5′-IMP	nd	0.142 ± 0.013 ^b^	0.090 ± 0.015 ^a^	nd	nd	nd	nd
Total 5′-nucleotides		0.010 ± 0.000	0.404 ± 0.051	0.311 ± 0.047	0.031 ± 0.002	0.033 ± 0.005	0.025 ± 0.016	0.036 ± 0.018
	oxalic acid	0.320 ± 0.020 ^d^	0.727 ± 0.010 ^e^	0.883 ± 0.037 ^f^	0.144 ± 0.054 ^c^	0.107 ± 0.012 ^b,c^	0.076 ± 0.004 ^a,b^	0.054 ± 0.012 ^a^
	ascorbic acid	nd	0.003 ± 0.000 ^a^	0.020 ± 0.000 ^b^	nd	nd	nd	nd
	lactic acid	nd	0.607 ± 0.017 ^c^	0.803 ± 0.005 ^d^	0.020 ± 0.005 ^a^	0.198 ± 0.004 ^b^	0.017 ± 0.001 ^a^	nd
	citric acid	0.020 ± 0.000 ^b^	0.010 ± 0.002 ^a^	0.070 ± 0.004 ^d^	0.063 ± 0.006 ^c^	nd	nd	nd
	succinic acid	0.400 ± 0.070 ^bc^	2.637 ± 0.037 ^e^	4.580 ± 0.090 ^f^	0.388 ± 0.076 ^b^	0.679 ± 0.070 ^d^	0.475 ± 0.018 ^c^	0.132 ± 0.016 ^a^
Total organic acids		0.740 ± 0.090	3.984 ± 0.066	6.356 ± 0.136	0.615 ± 0.141	0.984 ± 0.086	0.568 ± 0.023	0.186 ± 0.028

Note: nd means not detected; different superscripts represent significant differences between samples (*p* < 0.05); SP, stewed pepper; SS-S, soup sample in stewed sheep tail fat; SSP-S, soup sample in stewed sheep tail fat with prickly ash addition; SS-B, fat block sample in stewed sheep tail fat; SSP-B, fat block sample in stewed sheep tail fat with prickly ash addition; SS-O, upper oil sample in stewed sheep tail fat; SSP-O, upper oil sample in stewed sheep tail fat with prickly ash addition.

## Data Availability

The data presented in this study are available on request from the corresponding author.

## Supplementary Materials

The following supporting information can be downloaded at: https://www.mdpi.com/article/10.3390/foods11070896/s1, Figure S1: Single factor optimization results, Figure S2: The proportion of three types of amino acids in stewed sheep tail fat samples, Figure S3: The EUC value of samples, Table S1: Factors and conditions for optimizing stewed sheep tail fat samples, Table S2: Taste evaluation with compounds added to taste threshold solutions.
