# Supplementary material for: Characterization of Taste Compounds and Sensory Evaluation of Soup Cooked with Sheep Tail Fat and Prickly Ash
Source: Foods. 2022 Mar 22;11(7):896. doi: 10.3390/foods11070896 (PMC8997404; doi:10.3390/foods11070896)
Supplement: Supplementary file 1 [file foods-11-00896-s001.zip › foods-1613120-supplementary.pdf]

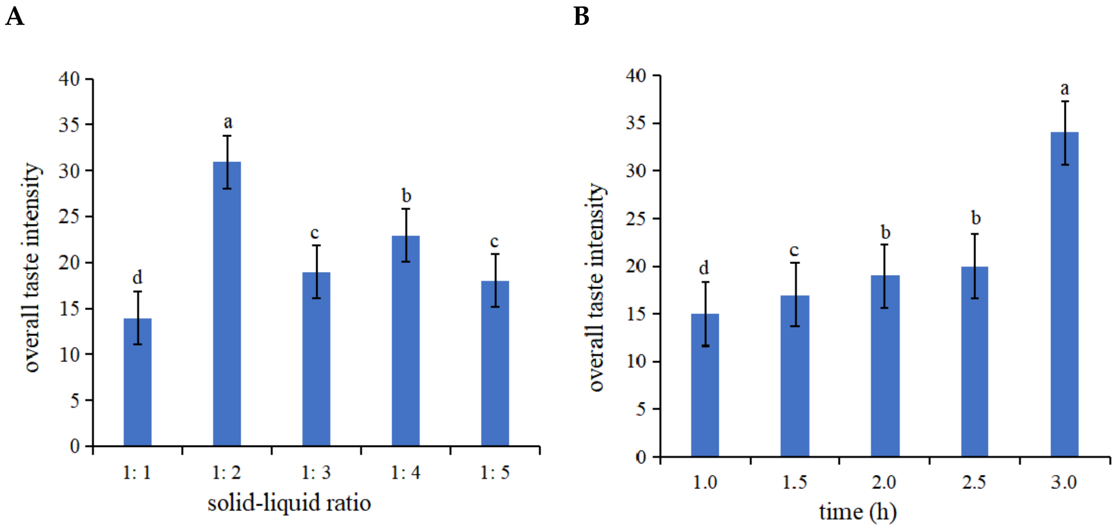

Figure S1. Sngle factor optimization results. (**A**, overall taste intensity of solid-liquid ratio; **B**, overall taste intensity of stewing time). Letters (a,b,c,d) indicate significantly difference at $p < 0.05$ (Duncan test).

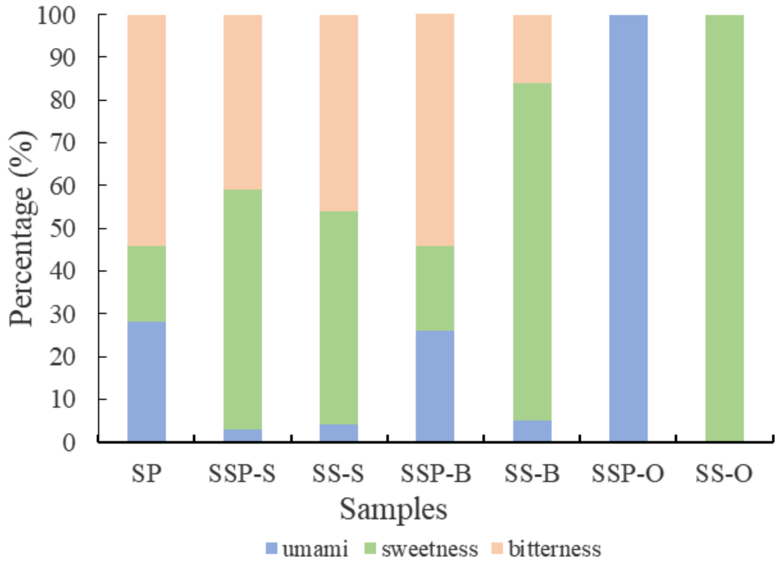

Figure S2. The proportion of three types of amino acids in stewed sheep tail fat samples.

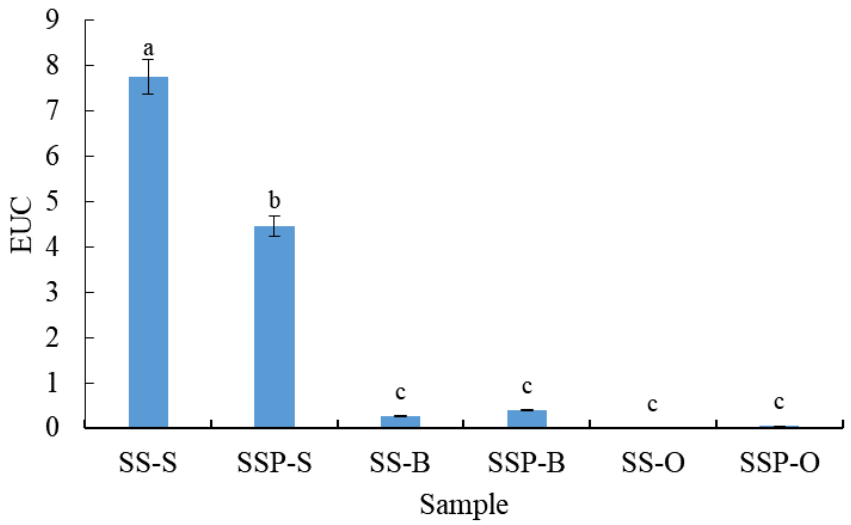

Figure S3. The EUC value of samples. Letters (a,b,c) indicate significantly difference at $p < 0.05$ (Duncan test).

Table S1. Factors and conditions for optimizing stewed sheep tail fat samples.

Factors	Other conditions
Solid-liquid ratio	100 g sheep tail oil with the solid-liquid ratio of 1: 1, 1: 2, 1: 3, 1: 4 and 1: 5, respectively, with stewing time 2 h.
Stew time	100 g sheep tail oil and 200 g with the stewing time of 1.0 h, 1.5 h, 2.0 h, 2.5 h and 3.0 h, respectively.
Spices selection	100 g sheep tail oil, 200 g water in an electric stew pan, and 0.2% (percentage of the total weight of sheep tail oil and water) spices in different kinds was added, namely ginger, cassia, nutmeg, white pepper and pepper, respectively, with stewing time 3.0 h.
Addition of optimized spice	100 g sheep tail oil, 200 g water with optimized spice addition of 0.1%, 0.2%, 0.3%, 0.4% and 0.5% (percentage of the total weight of sheep tail oil and water), respectively, with stewing time 3.0 h.

Table S2. Taste evaluation with compounds added to taste threshold solutions.

Name	Concentration (mg/L)				
	1times	2times	5times	7times	10times
Thr	153.61	307.22	768.05	1075.27	1536.10
Asp	3.99	7.98	19.95	27.93	39.90
lactic acid	3.29	6.58	16.45	23.03	32.90
citric acid	0.93	1.86	4.65	6.51	9.30
succinic acid	31.10	62.20	155.50	217.70	311.10
